# Gender-specific outcomes in immune checkpoint inhibitor therapy for advanced or metastatic urothelial cancer: a systematic review and meta-analysis

**DOI:** 10.1007/s00432-023-04788-x

**Published:** 2023-04-20

**Authors:** Laila Schneidewind, Bernhard Kiss, Friedemann Zengerling, Angelika Borkowetz, Sebastian Graf, Jennifer Kranz, Desiree L. Dräger, Annabel Graser, Laura Bellut, Annemarie Uhlig

**Affiliations:** 1 Department of Urology, University Medical Center Rostock, Rostock, Germany; 2 Department of Urology, University Hospital of Bern, Bern, Switzerland; 3 Department of Urology and Pediatric Urology, University Hospital Ulm, Ulm, Germany; 4 Department of Urology, Technische Universität Dresden, Dresden, Germany; 5 Department of Urology and Andrology, Kepler University Hospital Linz, Linz, Austria; 6 Department of Urology and Pediatric Urology, University Medical Center RWTH Aachen, Aachen, Germany; 7 Department of Urology and Kidney Transplantation, Martin Luther University, Halle (Saale), Germany; 8 Department of Urology, Ludwig Maximilian University, Munich, Germany; 9 Department of Urology and Pediatric Urology, University Hospital Erlangen, Erlangen, Germany; 10 Department of Urology, University Medical Center Göttingen, Göttingen, Germany; 11 Department of Urology, University Hospital Rostock, Ernst-Heydemann-Str. 6, 18055 Rostock, Germany

**Keywords:** Bladder cancer, Urothelial cancer, Immunotherapy, Gender, Overall survival

## Abstract

**Purpose:**

To analyze gender-specific differences in survival parameters in advanced or metastatic urothelial cancer patients undergoing immune checkpoint inhibition.

**Methods:**

The primary aim of this systematic review and meta-analysis was to evaluate gender-specific differences in disease-free (DFS), progression-free (PFS), cancer-specific survival (CSS), event-free survival (EFS), overall survival (OS) and objective response rate (ORR). The sources MEDLINE, Embase and Cochrane Library were systematically searched from January 2010 to June 2022. No restrictions were made concerning language, study region or publication type. A comparison of gender-specific differences in survival parameters was performed using a random-effects meta-analysis. A risk of bias assessment was done using the ROBINS-I tool.

**Results:**

Five studies were included. In a random-effect meta-analysis of the studies, PCD4989g and IMvigor 211 with both using atezolizumab, females were more likely to have better objective response rate (ORR) than men (OR 2.24; 95% CI 1.20–4.16; *p* = 0.0110). In addition, females had a comparable median OS to men (MD 1.16; 95% CI − 3.15–5.46; *p* = 0.598). In summary, comparing all results, a tendency was seen toward better response rates and survival parameters in female patients. The risk of bias assessment yielded an overall low risk of bias.

**Conclusions:**

There is a tendency toward better outcomes in women for immunotherapy in advanced or metastatic urothelial cancer, but only for the antibody atezolizumab women have a significantly better ORR. Unfortunately, many studies fail to report gender-specific outcomes. Therefore, further research is essential when aiming for individualized medicine. This research should address immunological confounders.

**Supplementary Information:**

The online version contains supplementary material available at 10.1007/s00432-023-04788-x.

## Introduction

Following the 2018 GLOBOCAN data, urothelial carcinoma of the bladder is the tenth most common malignancy worldwide, with 549,393 new cases and 200,000 cancer-related deaths. In the USA, bladder cancer comprises 5% of new cancer diagnoses and is the sixth most prevalent malignancy. Approximately, 75% of the newly diagnosed patients have non-muscle-invasive bladder cancer (tumor that spreads to the mucosa [carcinoma in situ, Ta] and lamina propria [stage T1]), while the remaining 25% of the patients have muscle-invasive carcinoma (tumor invasion to the muscle layer of the bladder; stage T2 and beyond). Prognosis depends on the type of bladder cancer, with 5-year rates ranging from 96% for non-muscle-invasive bladder cancer to 5% for metastatic cases. An estimated 17,240 deaths were caused by bladder cancer in the USA in 2018 (Bray et al. 2018; Burger et al. 2013; European Association of Urology (EAU) guidelines on muscle-invasive and metastatic bladder cancer 2022).

Furthermore, immune checkpoint inhibitor therapy has a rising relevance in bladder cancer, especially in advanced and metastatic disease (European Association of Urology (EAU) guidelines on muscle-invasive and metastatic bladder cancer 2022; Tran et al. 2021). There are hints from clinical trials and literature that there are significant differences in therapy responses between men and women (Otto et al. 2012; Donsky et al. 2014; Mungan et al. 2000; Uhlig et al. 2018). Unfortunately, data about gender-specific differences in immunotherapy in metastatic or advanced disease are sparse and inconsistent.

Consequently, the primary aim of this systematic review and meta-analysis was to evaluate gender-specific differences in disease-free (DFS), progression-free (PFS), cancer-specific survival (CSS), event-free survival (EFS) and overall survival (OS) in those patients and objective response rate (ORR). The secondary aims are gender-specific differences in adverse events and quality of life (QoL). According to the PICO (Patient, Intervention, Comparison and Outcome), we included patients with metastatic or advanced urothelial carcinoma receiving immune checkpoint inhibitor therapy and we compared survival parameters regarding the gender of these patients.

## Materials and methods

### Search strategy

In June 2022, we performed a systematic literature search using MEDLINE via PubMed, Embase and the Cochrane Library. The search algorithm broadly included the search term clusters gender, cystectomy, bladder cancer, immunotherapy and survival. The supplementary material (Supplementary 1) details the complete search algorithms. Reference lists of included articles, as well as review articles, were searched to identify additional records. No restrictions were made concerning language, study region or publication type. Publication date was included after January 2010 because immune checkpoint inhibition therapy was not established before. This study was prospectively registered at PROSPERO (https://www.crd.york.ac.uk/prospero/; ID CRD 42022308399).

### Study inclusion and exclusion criteria

The predefined primary outcomes were gender-specific differences in DFS, PFS, CSS, EFS and OS as well as ORR following mono-immunotherapy for metastatic or advanced bladder cancer. We included only randomized controlled trials (RCTs). Combination therapies with radiotherapy, chemotherapy or other targeted therapies were excluded. Additionally, neoadjuvant therapies prior to radical cystectomy and urinary diversion were also excluded since this surgical treatment is known to have immunological effects. The larger and more comprehensive publication was included if more than one publication evaluated the same patient cohort.

### Data extraction

An a priori defined standardized data extraction process was used for every included record. Extracted variables included author(s), year of publication, study country, population size, percent of female patients, cancer stage and grade, histopathological cancer subtype, length of follow-up, details on immunotherapy, variables adjusted for in multivariable Cox regression models and HR or OR measures with the associated 95% CI for DFS, PFS, CSS, EFS and OS as well as ORR. Furthermore, adverse event rates as well as all available quality of life data were extracted. Study extraction was independently performed by two review authors. Inconsistencies were resolved by a third review author. The online platform covidence (https://www.covidence.org/; Veritas Health Innovation Ltd, Melbourne, Australia) was used for the screening and data extraction process.

### Study quality assessment

Two reviewers independently assessed the risk of bias with the ROBINS-I-tool (Cochrane Germany 2021). This tool includes seven domains of bias: risk of bias due to confounding, bias in the selection of participants into the study, bias in classification of interventions, bias due to deviations from intended interventions, bias due to missing data, bias in the measurement of outcomes and bias in the selection of the reported results for one outcome measurement. The domains are combined to an overall risk of bias. Any disagreements were resolved by the involvement of a third review author.

### Statistical analysis

Comparison of gender-specific differences in survival parameters was performed using a random-effects meta-analysis with the Mantel–Haenszel method (here for ORR) and the inverse variance method weighting for pooling of continuous outcome data (here for median OS) to account for clinical heterogeneity (Hakulinen 1981; Shu et al. 2021). In all provided analyses, male patients were considered the referent. Studies providing estimates with a female referent were back-calculated by inversing the hazard ratios (HR) and the associated confidence intervals (CIs). Between studies, heterogeneity was assessed by the I^2^ statistic with the associated 95% CI, the Chi-square *p* values of heterogeneity and visual inspection of forest plots. Heterogeneity was interpreted as limited:—*I*^2^ = 0–40%, moderate—*I*^2^ = 41–60%, substantial—*I*^2^ = 61–80% and considerable *I*^2^ = 81—100%. All statistical analyses were performed with R version 4.2.1 (https://www.r-project.org/) and RStudio (RStudio, Boston, Massachusetts) and the R package meta (Schwarzer 2007). The alpha level indicating statistical significance was predefined as 0.05 for all analyses except the assessment of heterogeneity, which was considered at alpha = 0.1. All provided *p* values are two sided.

## Results

### Study characteristics

**Fig. 1:**
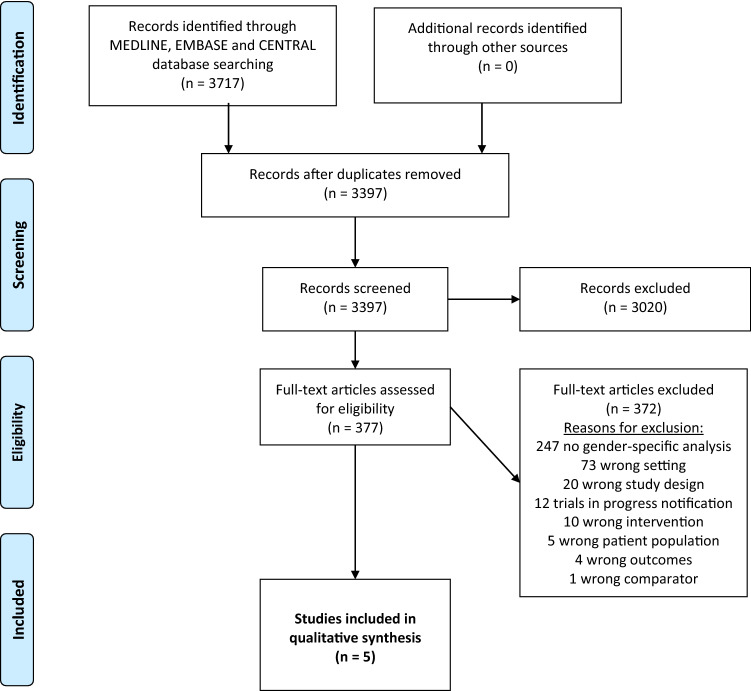
PRISMA flowchart

Of the 3717 studies identified by systematic literature search, 5 fulfilled the inclusion criteria. Figure 1 shows the PRISMA (Preferred Reporting Items for Systematic Reviews and Meta-Analyses) flowchart of the study selection process (Liberati et al. 2009). Furthermore, Table 1 summarizes the characteristics of the included studies (Aragaki et al. 2022; Bajorin et al. 2021; Bellmunt et al. 2017, 2021; Hoffman-Censits et al. 2019). All included studies were performed in a multi-centric, multi-national setting and published in an English language journal. Additionally, the included studies' median follow-up differs considerably, ranging from 14.1 to 21.9 months. The percentage of included females ranged from 21.1 to 26.4%. Interestingly, all studies included urothelial carcinoma of the upper tract (UTUC). The proportion of UTUC in the study population ranged between 6.7 and 21.0%. Two studies evaluated immunotherapy in an adjuvant setting for advanced or high-risk urothelial cancer (UC), one phase III study for nivolumab (Bajorin et al. 2021) and one phase III study for atezolizumab (Bellmunt et al. 2021). The three remaining studies addressed advanced or metastatic disease (Aragaki et al. 2022; Bellmunt et al. 2017; Hoffman-Censits et al. 2019). In summary, three studies investigated atezolizumab, one study nivolumab and one pembrolizumab. As well as the primary end point for three studies was OS, and for two studies DFS, so the studies’ settings were too heterogeneous to perform a meta-analysis in most cases (Aragaki et al. 2022; Bajorin et al. 2021; Bellmunt et al. 2017, 2021; Hoffman-Censits et al. 2019). In detail, no pooling was possible for DFS, PFS, CSS and EFS.

**Table 1:** Summary of study characteristics (*n* = 5)

Reference	Trial numbers	Setting	Study region	Upper urinary tract carcinoma included	Neoadjuvant pretreatment allowed	Radiation pretreatment allowed	Drug	Sample size; % female	Survival end point for gender analysis	Follow-up period	Quality of life data included	Special features
Aragaki et al. (2022)	IMvigor 210, 406 patients from TCGA MIBC	Phase II, metastatic and advanced disease, multi-centric	North America	Yes	No	Yes	Atezolizumab	406; 26.4 (for TCGA data)	OS	No detailed information in publication	No	Detailed analysis for gender-specific biomarkers
Bajorin et al. (2021)	CheckMate 275	Phase III, adjuvant for advanced and high-risk, multi-centric	North and South America, Europe, Asia, Australia	Yes	Yes	No	Nivolumab	709; 23.8 (analysis with 353 with 24.9% females)	DFS; RFS and recurrence rates	Median 20.9 months	Yes	–
Bellmunt et al. (2017)	Keynote-045	Phase III, metastatic and advanced, second-line, multi-centric	North America, Europe	Yes	Yes	No	Pembrolizumab	542; 25.8	OS; death rates	Median 14.1 months	No	–
Bellmunt et al. (2021)	IMvigor 010	Phase III, adjuvant for advanced and high-risk, multi-centric	North America	Yes	Yes	No	Atezolizumab	809; 21.1 (analysis with 406 with 20.7% females)	Median DFS disease rates	Median 21.9 months	No	–
Hoffman-Censits et al. (2019) [Abstract]	PCD4989g, IMvigor 210, IMvigor 211, SAUL	Pooled Analysis, locally advanced and metastatic disease, multi-centric	Worldwide	Yes	Yes	No	Atezolizumab	1995; 22.6 pooled analysis for: A) PCD4989g: 95; 24.2 B) IMvigor 211 atezo: 467; 23.6	Median OS, ORR; disease rates	Different among included studies	No	–

However, Bajorin et al. reported an HR for nivolumab versus placebo of 0.76 (95% CI 0.50–1.16) for disease recurrence or death. Bellmunt et al. 2017 described an HR for pembrolizumab versus conventional chemotherapy of 0.78 (95% CI 0.49–1.24) for OS and Bellmunt et al. 2021 an HR for atezolizumab versus observation 1.00 (95% CI 0.65–1.52) for DFS all for female patients, respectively (Bajorin et al. 2021; Bellmunt et al. 2017, 2021).

### Pooled analysis for primary outcomes ORR and median OS

**Fig. 2:**
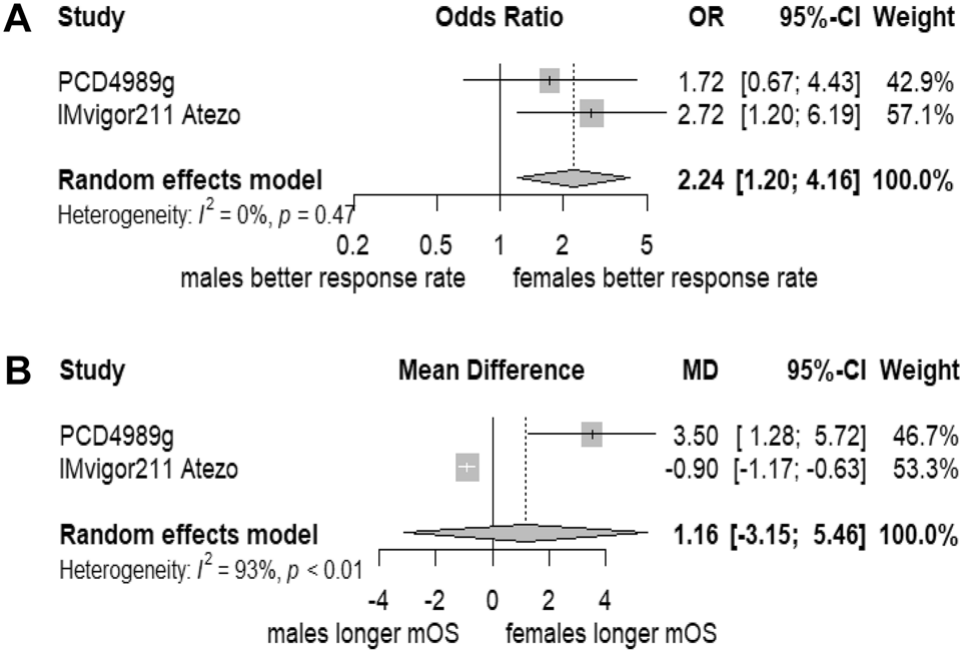
Forest plot of the random-effect meta-analysis. **A** Random-effect meta-analysis (Mantel–Haenszel method) for ORR. **B** Random-effect meta-analysis (inverse variance method) for median OS

In a random-effect meta-analysis of the studies, PCD4989g and IMvigor 211 atezolizumab included in the pooled study published by Hoffman-Censits et al. (2019), females were more likely to have better objective response rate (ORR) than men (OR 2.24; 95% CI 1.20–4.16; *p* = 0.0110). In addition, females have a comparable median OS to men (MD 1.16; 95% CI − 3.15–5.46; *p* = 0.598). A forest plot showing the random-effect meta-analysis is provided in Fig. 2. For ORR, there was a limited heterogeneity (*I*^2^ = 0%; *p* = 0.47), while for median OS, there was a statistically significant considerable heterogeneity (*I*^2^ = 93.2%; 95% CI 77.9–98.0%; *p* = 0.0001). Interestingly, both pooled studies in investigated the antibody atezolizumab.

As described above, no further pooling was possible. Nevertheless, comparing the results, a tendency was seen toward better response rates and survival parameters in female patients receiving immunotherapy for advanced or metastatic urothelial carcinoma.

Due to the small number of included studies, no subgroup or sensitivity analyses were possible. Likewise, we did not perform any analyses for publication bias.

### Secondary outcomes: adverse events and QoL

Three studies reported adverse events of immunotherapy but without any gender-specific analysis. On the whole, adverse events of all grades in the immunotherapy group ranged from 60.9 to 77.5% (Bajorin et al. 2021; Bellmunt et al. 2017, 2021).

Only one study published QoL data without gender-specific data (Bajorin et al. 2021).

### Quality assessment

**Fig. 3:**
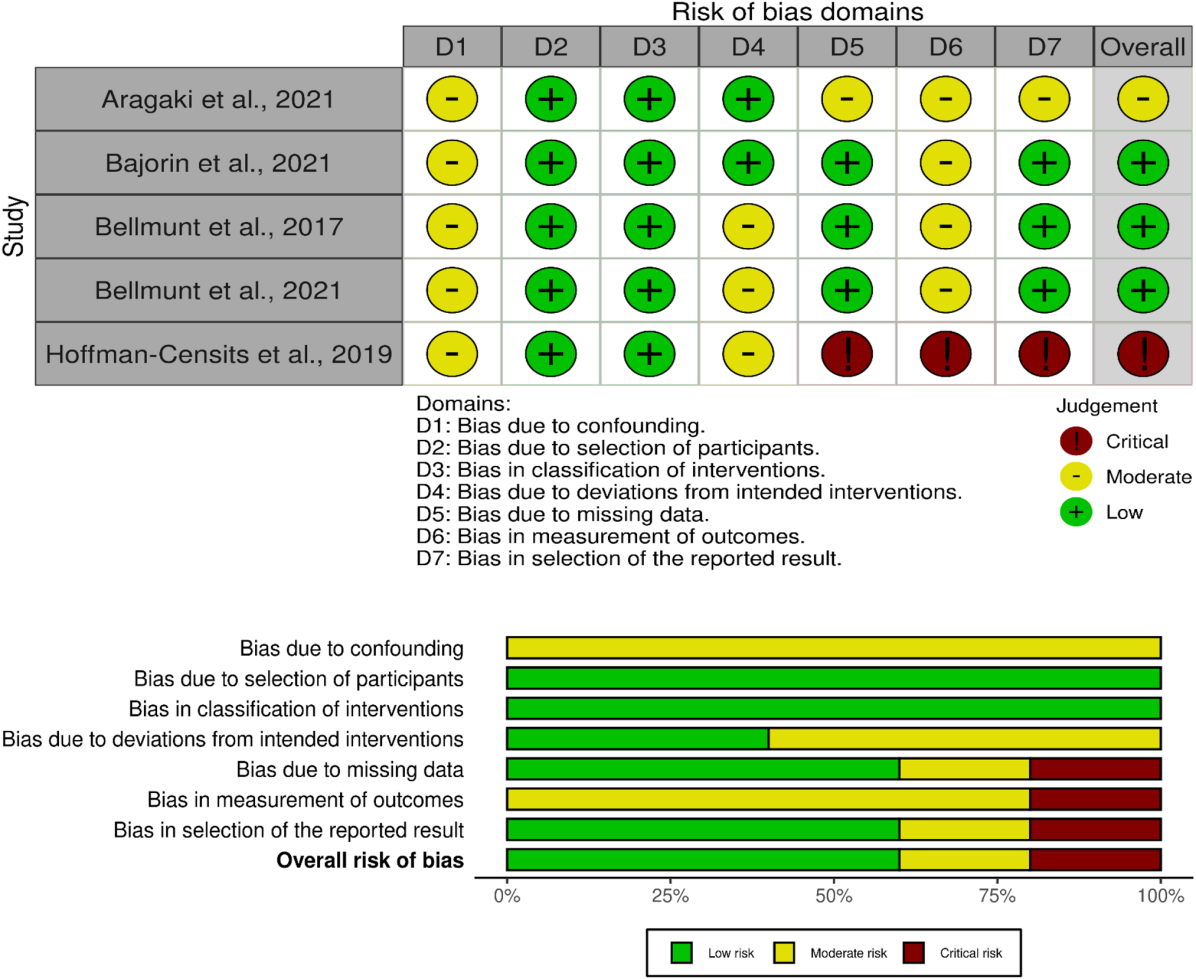
Detailed risk of bias assessment

The risk of bias assessment yielded an overall low risk of bias. The reason for limited quality or serious risk of bias in one study was mainly because this was a conference abstract (Hoffman-Censits et al. 2019). Additionally, in all studies, there was a moderate risk for bias due to confounding, since there were a number of immunological confounders known in immunotherapy, which were not all adjusted for (Aragaki et al. 2022; Bajorin et al. 2021; Bellmunt et al. 2017, 2021; Hoffman-Censits et al. 2019). Table 2 gives an overview of the risk of bias assessment. Furthermore, Fig. 3 details the risk of bias evaluation.

**Table 2:** Risk of bias assessment with ROBINS-I (*n* = 5)

	Aragaki et al. (2022) (for OS)	Bajorin et al. (2021) (for DFS)	Bellmunt et al. (2017) (for OS)	Bellmunt et al. (2021) (for DFS)	Hoffman-Censits et al. (2019) (for OS; abstract)
Risk of bias due to confounding	Moderate	Moderate	Moderate	Moderate	Moderate
Bias in selection of participants into the study	Low	Low	Low	Low	Low
Bias in classification of interventions	Low	Low	Low	Low	Moderate
Bias due to deviations from intended interventions	Low	Low	Moderate	Moderate	Moderate
Bias due to missing data	Moderate	Low	Low	Low	Serious
Bias in measurement of outcomes	Moderate	Moderate	Moderate	Moderate	Serious
Bias in selection of the reported results	Moderate	Low	Low	Low	Serious
Overall risk of Bias	Moderate	Low	Low	Low	Serious

### Other study results

Notably, Aragaki et al. analyzed also gender-specific biomarkers, especially based on the intramural expression of B cell gene signature. On the whole, the authors stated: tumors with high levels of B cell and CD8+ T cell gene signatures (BCGS/CD8TGS or B8T high/high) were associated with the longest OS of all B8T groups. Moreover, the B8T cell signature stratified patients whose tumors had a high tumor mutational burden or high programmed death ligand 1 (PD-L1) into subsets with differential OS outcomes. Whereas the B8T high/high tumors were associated with the best clinical outcomes in men treated with immunotherapy, they were not associated with better OS in women. Conversely, women with B8T high/high tumors had the best clinical outcomes in non–immunotherapy-treated muscle-invasive bladder cancer. Consequently, the authors concluded that the B8T signature can enhance OS stratification in patients with advanced urothelial carcinoma who are treated with immunotherapy and that sex-specific differences in the tumor immune microenvironment may drive disparate outcomes (Aragaki et al. 2022).

## Discussion

We performed a systematic review and meta-analysis about gender-specific differences in immunotherapy for advanced or metastatic urothelial carcinoma. Unfortunately, many studies did not report any gender-specific analyses, even though there are hints from the literature that gender-specific differences may relevantly impact therapeutic outcomes (Otto et al. 2012; Donsky et al. 2014; Mungan et al. 2000; Uhlig et al. 2018). Nevertheless, 247 studies were excluded during full-text screening due to missing analyses. However, we were able to perform a pooled analysis of two studies with the antibody atezolizumab with a tendency for a better outcome in women, which is a very interesting and surprising result, because most recent evaluations describe higher tumor stages and worse OS in advanced urothelial bladder cancer for women (Soave et al. 2015; Krimphove et al. 2021). This might be the same in our populations, especially for the TNM stage. Consequently, it must be discussed that men might have a worse response to the antibody atezolizumab in particular and maybe even to immunotherapy in general. Furthermore, men are overrepresented in all studies accounting for about 75% of the study populations due to the epidemiological nature of the disease.

In the context of the observed effects of atezolizumab, it must also be discussed that this antibody might play an essential role in response. It is known, that atezolizumab is not the ideal antibody for bladder cancer treatment, and the results are inferior compared to other approved drugs, except from the circulating tumor DNA studies from Powles et al. (Powles et al. 2021; Szabados et al. 2022).

What are the reasons for these substantial differences? It might be due to the different immune responses of men and women, which are already better described for infectious diseases (McClelland and Smith 2011). Moreover, this is a big advantage of our systematic review: we tried to eliminate most immunological confounders and establish a homogenous study group, e.g., by the exclusion of combination therapies, such as radiation, which can also have immunogenic effects. In detail, many immunological confounders can influence response rates to immunotherapy. The first confounder would be the surgery. It is known that radical cystectomy is a major intervention which results in post-aggression syndrome. This, on the one hand, can lead to immunosuppression, but on the other hand, due to trauma and cell death to immune activation with neoantigen expression (Gaudillière et al. 2014; Beger et al. 1981). Unfortunately, little is known about this post-aggression syndrome in radical cystectomy and even TUR-BT as bladder cancer surgeries (Beger et al. 1981). Additionally, there are also differences in radical cystectomy in the degree of trauma, such as the extent of lymphadenectomy, type of urinary diversion or open versus minimal-invasive approach. Second immunological confounders are the prior therapies before immunotherapy, and this is very heterogenous in the included studies, e.g., some allowed prior neoadjuvant platin-based chemotherapy and some allowed prior radiation (Aragaki et al. 2022; Bajorin et al. 2021; Bellmunt et al. 2017, 2021; Hoffman-Censits et al. 2019; see Table 1). The third important immunological confounders are the patient’s comorbidities and drug treatment, such as diabetes mellitus or malnutrition (Rosenthal and Moore 2015). In summary, many different variables can influence immune responses, so multi-center clinical studies should try to evaluate homogenous populations addressing these factors or consider them in multivariate adjustment when reporting results. This is also why it is challenging to conclude gender-specific differences in this setting, and all of the included studies here have a moderate risk of bias due to confounding factors.

However, the gender-specific differences in the immune response are even more important, so even one of the included studies in this systematic review reported sex-specific differences in the tumor microenvironment and the immune system (Aragaki et al. 2022). There is rising evidence that there are gender-specific cytokine pathways in the immune response to malignant disease, e.g., as Capone et al. concluded in their study. Thus, the sexual dimorphism of the immune signals, including IFN-1 ones, may be a new attractive perspective for optimizing immunotherapy. Moreover, this critical challenge could represent a future opportunity to better integrate immunotherapies with other conventional (cytotoxic) as well as targeted therapies (Berghella et al. 2017; Imhara et al. 2005; Capone et al. 2018). Additionally, gender-specific immune responses might also arise from epigenetic differences in men and women (Migliore et al. 2021). On the whole, further research in gender-specific immune responses to bladder cancer, especially immunotherapy, is essential for individualized optimal therapy. Mancini et al. summarized this issue best. There is a lack of evidence-based recommendations for gender-specific management of bladder cancer and this is an approach to individualized medicine. Future research should guarantee greater inclusion of women in trials and focus on improving the effectiveness of therapies in women, perhaps even exploring different therapeutic approaches in men and women (Mancini et al. 2020). In our opinion, the optimal inclusion of women in those studies is only possible in a multi-center setting.

Moreover, there are several factors in the included studies which can also influence gender-specific differences in the outcome parameters and should be addressed in further research, such as reporting the results only for the intention-to-treat population, the histological and molecular subtypes of urothelial carcinoma, the PD-1/PD-L1 expression status in the studies and their immune-histochemical evaluation with different assays as well as the proportion of bladder carcinomas and UTUC in the studies. In our mind, two things are most important. Firstly, the PD-1/PD-L1 expression, e.g., Eckstein et al. reviewed and summarized the problems of that issue, so many aspects of PD-L1 immunohistochemistry in advanced urothelial carcinoma remain unclear and unfinished and should be refined, such as more specific data on tumor heterogeneity, cutoff values and tumor cell immunohistochemistry are needed to guide the pathologist to optimal scoring and so the clinicians for optimal treatment. Furthermore, the authors suggest the combination of PD-L1 with other new biomarkers, such as tumor mutational burden or immune cell infiltration, will be required for an optimal personalized patient´s selection, which might improve the outcomes (even gender-specific ones) (Eckstein et al. 2019). Secondly, the proportion of bladder and upper tract carcinomas because there is evidence that patients with UTUC have lower PD-L1 expression than those with bladder urothelial carcinoma and they both exhibit significant differences in the prevalence of genomic landscape and carcinogenesis (Yang et al. 2021). Consequently, this might also differ in the sexes, but detailed evaluations are missing.

In addition, we aimed to report gender-specific differences in adverse events and QoL as secondary outcomes of our meta-analysis. Unfortunately, there was no gender-specific analysis of this data in the included studies, and only one study reported QoL data (Bajorin et al. 2021). Still, there might be gender-specific differences here as well. Jehn et al. concluded that interventions during oral cancer therapy should address psychological variables and have gender-specific elements to improve health-related QoL (HRQoL) after treatment (Jehn et al. 2022). Interestingly, gender-specific differences for HRQoL were also described for larynx carcinoma (Tan et al. 2016). This explicit knowledge about gender-specific adverse events, HRQoL and psychological aspects is very important to improve treatment strategies and other approaches to individualized medicine. Wessels et al. put it this way: gender impacts cancer patients´ needs and preferences and should be considered for optimal cancer care. Additionally, cancer care might be tailored toward gender, e.g., with regard to the means and extent of communication, manner and extent of support, counseling and rehabilitation, consultation length and physician’s assignment (Wessels et al. 2010).

Our study has several limitations such as the small number of included studies, only one pooled analysis with the antibody atezolizumab, and the impossibility of performing subgroup analysis as we proposed in our initial review protocol. In our opinion, subgroup analysis or subsets in gender-specific questions would be of particular value for further research, e.g., for urothelial bladder cancer versus UTUC, for the study region, for the risk of bias, especially publication bias, and abstracts versus full publications. Additionally, a vast majority of studies had to be excluded due to the fact that they did not report gender-specific differences, which is absolutely warranted in further research and important for clinical practice.

Nevertheless, our meta-analysis is the first to evaluate gender-specific differences in immune checkpoint inhibitor therapy for advanced or metastatic urothelial cancer. We performed a rigorous literature search and presented as homogeneous as possible data treatment group data.

## Conclusions

There is a tendency toward better outcomes in women for immunotherapy in advanced or metastatic urothelial cancer, but only for the antibody atezolizumab women have a significantly better ORR. Unfortunately, many studies fail to report gender-specific differences, especially regarding adverse events or HRQoL. Therefore, further research is essential when aiming for individualized medicine. This research should address immunological confounders, gender-specific differences in the immune response to cancer and immunotherapy as well as epigenetics, differences in PD-L1 expression and other histological or molecular biomarkers and HRQoL including gender-specific psychological aspects and treatment needs.

## Supplementary Information

Below is the link to the electronic supplementary material.

Supplementary file1 (DOCX 22 KB)

## Data Availability

The dataset generated during this systematic review is available from the corresponding author on reasonable request.
