# Supplementary material for: Gender-specific outcomes in immune checkpoint inhibitor therapy for advanced or metastatic urothelial cancer: a systematic review and meta-analysis
Source: J Cancer Res Clin Oncol. 2023 Apr 20;149(11):9399–408. doi: 10.1007/s00432-023-04788-x (PMC10374671; doi:10.1007/s00432-023-04788-x)
Supplement: Supplementary file 1 — Supplementary file1 (DOCX 22 KB) [file 432_2023_4788_MOESM1_ESM.docx]

**Supplementary 1 – Search Strategies**

**MEDLINE via PubMed**

(“Urinary Bladder Neoplasms”[Mesh] OR “Carcinoma, Transitional Cell”[Mesh] OR (bladder cancer*) OR (bladder tumor*) OR (bladder tumour*) OR (bladder neoplas*) OR (urothelial cancer*) OR (urothelial tumor*) OR (urothelial tumour) OR (urothelial neoplas*) OR (transitional cell carcinoma))

AND

((checkpoint inhibitor*) OR (pd-1) OR (programmed death 1) OR (pd-l1) OR (cytotoxic t-lymphocyte-associated protein 4) OR (cytotoxic t lymphocyte-associated antigen 4) OR (ctla-4) OR (atezolizumab) OR (avelumab) OR (durvalumab) OR (nivolumab) OR (pembrolizumab))

AND

(“Recurrence*”[Mesh] OR “Disease Progression”[Mesh] OR “Treatment Outcome*”[Mesh] OR “Survival”[Mesh] OR “Survival Analysis”[Mesh] OR (disease progression) OR (survival))

AND

(“Female*”[Mesh] OR “Male*”[Mesh] OR “Sex”[Mesh] OR “Women”[Mesh] OR “Men”[Mesh] OR (gender) OR (man) OR (men) OR (woman) OR (women) OR (male*) OR (female*) OR (sex))

**Embase**

| 1 | exp bladder tumor/ |
| --- | --- |
| 2 | exp transitional cell carcinoma/ |
| 3 | ((bladder or transitional cell or urothelial) adj1 (cancer* or carcinoma* or tumo?r*)).tw,kw. |
| 4 | exp immune checkpoint inhibitor/ |
| 5 | exp programmed death 1 ligand 1/ |
| 6 | cytotoxic T lymphocyte antigen 4/ |
| 7 | exp atezolizumab/ |
| 8 | exp avelumab/ |
| 9 | exp durvalumab/ |
| 10 | exp nivolumab/ |
| 11 | exp pembrolizumab/ |
| 12 | (checkpoint inhibitor* or pd-1 or programmed death 1 or pd-l1 or cytotoxic t-lymphocyte-associated protein 4 or cytotoxic t lymphocyte-associated antigen 4 or ctla-4 or atezolizumab or avelumab or durvalumab or nivolumab or pembrolizumab).mp. |
| 13 | exp recurrent disease/ |
| 14 | exp treatment outcome/ |
| 15 | exp survival/ |
| 16 | exp survival analysis/ |
| 17 | (Recurrence* or Disease Progression or treatment outcome* or survival).tw,kw. |
| 18 | exp female/ |
| 19 | exp male/ |
| 20 | exp sex/ |
| 21 | (gender or m#n or wom#n or male* or female* or sex).tw,kw. |
| 22 | 1 or 2 or 3 |
| 23 | 4 or 5 or 6 or 7 or 8 or 9 or 10 or 11 or 12 |
| 24 | 13 or 14 or 15 or 16 or 17 |
| 25 | 18 or 19 or 20 or 21 |
| 26 | 22 and 23 and 24 and 25 |

**Cochrane Library**

ID Search Hits

#1 [mh "Urinary Bladder Neoplasms"] 1609

#2 [mh "Carcinoma, Transitional Cell"] 622

#3 urothelial cancer*:ti,ab,kw 1117

#4 urinary bladder cancer*:ti,ab,kw 2297

#5 transitional cell carcinoma:ti,ab,kw 1409

#6 bladder cancer*:ti,ab,kw 5017

#7 bladder tumor*:ti,ab,kw 2406

#8 bladder tumour*:ti,ab,kw 656

#9 bladder neoplas*:ti,ab,kw 2686

#10 urothelial cancer:ti,ab,kw 1111

#11 urothelial tumor*:ti,ab,kw 686

#12 urothelial neoplas*:ti,ab,kw 415

#13 #1 or #2 or #3 or #4 or #5 or #6 or #7 or #8 or #9 or #10 or #11 or #12 6353

#14 [mh "Immune Checkpoint Inhibitors"] 70

#15 checkpoint inhibitor*:ti,ab,kw 1785

#16 [mh "Programmed Death Ligand 1"] 129

#17 pd-1:ti,ab,kw 2586

#18 programmed death 1:ti,ab,kw 1869

#19 pd-l1:ti,ab,kw 3116

#20 cytotoxic t-lymphocyte-associated protein 4:ti,ab,kw 64

#21 cytotoxic tlymphocyte-associated antigen 4:ti,ab,kw 4

#22 ctla-4:ti,ab,kw 588

#23 atezolizumab:ti,ab,kw 1118

#24 avelumab:ti,ab,kw 304

#25 durvalumab:ti,ab,kw 804

#26 [mh "nivolumab"] 583

#27 nivolumab:ti,ab,kw 2326

#28 pembrolizumab:ti,ab,kw 2296

#29 (#14 or #15 or #16 or #17 or #18 or #19 or #20 or #21 or #22 or #23 or #24 or #25 or #26 or #27 or #28) 8605

#30 [mh "recurrence"] 12761

#31 Recurrence*:ti,ab,kw 55795

#32 [mh "Disease Progression"] 7917

#33 Disease Progression:ti,ab,kw 50523

#34 [mh "Treatment Outcome"] 151827

#35 Treatment Outcome*:ti,ab,kw 410799

#36 [mh "Survival"] 134

#37 [mh "Survival Analysis"] 21860

#38 [mh "Disease-Free Survival"] 7175

#39 survival:ti,ab,kw 117174

#40 #30 or #31 or #32 or #33 or #34 or #35 or #36 or #37 or #38 or #39 519561

#41 [mh "female"] 480656

#42 female*:ti,ab,kw 823278

#43 [mh "male"] 459771

#44 male*:ti,ab,kw 779628

#45 [mh "sex"] 35

#46 sex:ti,ab,kw 55717

#47 [mh "women"] 832

#48 women:ti,ab,kw 166535

#49 woman:ti,ab,kw 166535

#50 [mh "men"] 62

#51 men:ti,ab,kw 85552

#52 man:ti,ab,kw 81661

#53 [mh "gender"] 272

#54 gender:ti,ab,kw 35597

#55 #41 or #42 or #43 or #44 or #45 or #46 or #47 or #48 or #49 or #50 or #51 or #52 or #53 or #54 998553

#56 #13 and #29 and #40 and #55
